# Association between *Taenia solium* infection and HIV/AIDS in northern Tanzania: a matched cross sectional-study

**DOI:** 10.1186/s40249-016-0209-7

**Published:** 2016-12-01

**Authors:** Veronika Schmidt, Christian Kositz, Karl-Heinz Herbinger, Hélène Carabin, Bernard Ngowi, Ezra Naman, Patricia P. Wilkins, John Noh, William Matuja, Andrea Sylvia Winkler

**Affiliations:** 1 Department of Neurology, University Hospital, Klinikum rechts der Isar, Technical University Munich (TUM), Ismaninger Str. 22, 81675 Munich, Germany; 2 Department of Infectious Diseases and Tropical Medicine (DITM), Medical Center of the University of Munich, Munich, Germany; 3 Department of Biostatistics and Epidemiology, University of Oklahoma Health Sciences Center, Oklahoma City, USA; 4 Muhimbili Medical Research Centre, National Institute for Medical Research (NIMR), Dar es Salaam, Tanzania; 5 HIV Care and Treatment Centre, Haydom Lutheran Hospital, Haydom, Tanzania; 6 Division of Parasitic Diseases and Malaria, Center for Global Health, Centers for Disease Control and Prevention (CDC), Atlanta, USA; 7 Department of Neurology, Muhimbili University of Health and Allied Sciences, Dar es Salaam, Tanzania; 8 Centre of Global Health, University of Oslo, Oslo, Norway

**Keywords:** *Taenia solium*, Taeniosis, Cysticercosis, Neurocysticercosis, HIV, AIDS, Co-infection, Helminth, Tapeworm, Prevalence

## Abstract

**Background:**

The frequency of *Taenia solium*, a zoonotic helminth, is increasing in many countries of sub-Saharan Africa, where the prevalence of the human immunodeficiency virus (HIV) is also high. However, little is known about how these two infections interact. The aim of this study was to compare the proportion of HIV positive (+) and negative (−) individuals who are infected with *Taenia solium* (TSOL) and who present with clinical and neurological manifestations of cysticercosis (CC).

**Methods:**

In northern Tanzania, 170 HIV+ individuals and 170 HIV– controls matched for gender, age and village of origin were recruited. HIV staging and serological tests for TSOL antibodies (Ab) and antigen (Ag) were performed. Neurocysticercosis (NCC) was determined by computed tomography (CT) using standard diagnostic criteria. Neurological manifestations were confirmed by a standard neurological examination. In addition, demographic, clinical and neuroimaging data were collected. Further, CD4^+^ cell counts as well as information on highly active antiretroviral treatment (HAART) were noted.

**Results:**

No significant differences between HIV+ and HIV– individuals regarding the sero-prevalence of taeniosis-Ab (0.6% vs 1.2%), CC-Ab (2.4% vs 2.4%) and CC-Ag (0.6% vs 0.0%) were detected. A total of six NCC cases (3 HIV+ and 3 HIV–) were detected in the group of matched participants. Two individuals (1 HIV+ and 1 HIV–) presented with headaches as the main symptom for NCC, and four with asymptomatic NCC. Among the HIV+ group, TSOL was not associated with CD4^+^ cell counts, HAART duration or HIV stage.

**Conclusions:**

This study found lower prevalence of taeniosis, CC and NCC than had been reported in the region to date. This low level of infection may have resulted in an inability to find cross-sectional associations between HIV status and TSOL infection or NCC. Larger sample sizes will be required in future studies conducted in that area to conclude if HIV influences the way NCC manifests itself.

**Electronic supplementary material:**

The online version of this article (doi:10.1186/s40249-016-0209-7) contains supplementary material, which is available to authorized users.

## Multilingual abstracts

Please see Additional file 1 for translations of the abstract into the six official working languages of the United Nations.

## Background

*Taenia solium* is a zoonotic parasite which has considerable impact on human and animal health as well as on the agricultural and health sectors in many low income countries [1]. In humans, the adult stage of the tapeworm is found in the intestines (taeniosis) and the larval stage can develop as cysticerci mainly in the subcutaneous tissue, skeletal and heart muscles (cysticercosis, CC), and of most concern for public health, in the brain (neurocysticercosis, NCC) [2–4].

NCC is believed to be the most common helminthic infection of the central nervous system (CNS) worldwide and is well known as a major cause of acquired epilepsy or epileptic seizures resulting in reduced quality of life, social stigma, and high care costs for affected individuals and their caretakers [5–7]. In areas where the infection is endemic, it is estimated that 30% of people with epilepsy (PWE) show lesions of NCC in their brain [8]. In a hospital-based cross-sectional study conducted in northern Tanzania in 2006, definitive and probable NCC (as classified by Del Brutto et al.) was found in 2.4 and 11.3% of PWE, respectively [9, 10]. In Zambia, in a cross-sectional community-based study among PWE, 4.1% could be revealed as definitive NCC and 24.5% as suggestive NCC. In the same study, 2.5 and 0.0% of controls in the non-PWE group were defined as definite and suggestive NCC [11]. Besides epilepsy, other clinical manifestations such as acute and chronic headaches, signs or symptoms of intracranial hypertension, neuropsychiatric disorders and focal neurological deficits have been described [6, 12].

Human infection by the adult tapeworm - *T. solium* taeniosis - causes only mild symptoms, such as abdominal pain or diarrhoea, if any [13]. Data on taeniosis prevalence in sub-Saharan Africa are still scarce. Cross-sectional studies conducted in rural communities have reported prevalence proportions of taeniosis using a copro-antigen-enzyme linked immunosorbent assay (copro-Ag-ELISA) of 19.9% in western Kenya, 6.3 to 11.9% in the Eastern Province of Zambia, and 1.1 to 5. 2% in Tanzania [14–18]. Moreover, a Tanzanian study conducted in Mbeya Rural District reported a taeniosis-antibody (Ab) prevalence of 4.1% using a rES38-immunoblot [17].

Many *T. solium* endemic areas in sub-Saharan Africa are also endemic for the human immunodeficiency virus (HIV) [19]. Nearly 25 million people are estimated to live with HIV/acquired immunodeficiency syndrome (AIDS) in sub-Saharan Africa [20]. While an overall decline in HIV/AIDS prevalence is observed in most African countries, the incidence of HIV is increasing in some rural areas [21] - most of them resource-poor - where *T. solium* has also been reported [12, 19]. This suggests the presence of co-infections in several predisposed countries. However, while HIV has been shown to interact with tuberculosis, malaria and some soil-transmitted parasitic infections [22–24], much less is known about how HIV modifies the manifestations of NCC. Not only may HIV modify clinical manifestations*,* but it may also impact the interpretation of sero-diagnostic results and required treatment schemes for NCC and taeniosis [25, 26]. Some authors have suggested that patients with higher CD4^+^ T-lymphocyte (CD4^+^) counts are more likely to develop symptomatic NCC needing treatment, whereas patients with advanced HIV and lower CD4^+^ counts present either asymptomatically or atypically (giant and racemose cysts) [27–30]. In contrast, in a cross-sectional study conducted among HIV+ people in Mozambique, no significant correlation was found between presence of CC-Ab and CD4^+^ counts [31]. The hypotheses that HIV+ individuals with low CD4^+^ counts are more likely to develop symptomatic NCC was not supported in another study [32]. A Mexican autopsy study showed that NCC was even less common in people with HIV (1.1%) than among people without HIV (2.4%) [33], whereas in South-Africa, NCC has been described as one of the most present focal brain lesions in people with HIV/AIDS presenting with neurological signs [30]. In addition to seemingly unpredictable NCC manifestations in people with HIV/AIDS, highly active antiretroviral therapy (HAART) may lead to activation of latent NCC in the context of an immune reconstitution inflammatory syndrome with potentially deleterious effects for the affected individual [32, 34].

Based on the above controversy, the aim of this study was to estimate the prevalence of and factors associated with *T. solium* infections (taeniosis, CC, and NCC) among HIV+ and HIV– individuals in northern Tanzania.

## Methods

### Study sites

**Fig. 1:**
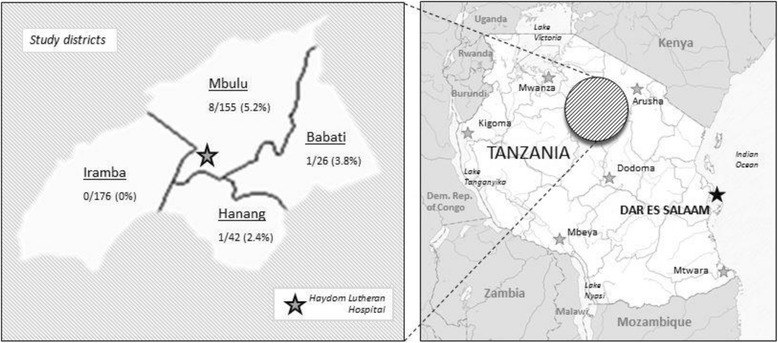
Map of study area and distribution of identified TSOL+ individuals of the initially recruited study population with known origin and serology (*n* = 399)

HIV positive (HIV+) individuals were recruited at the HIV Care and Treatment Centre (CTC) of HLH in Haydom, Mbulu district, Manyara region, northern Tanzania. HLH is a 429 bed-hospital serving around 15 400 inpatients and 70 150 outpatients per year [35]. As of December 2012, 1 898 HIV patients had sought care at the CTC clinic. All HIV/AIDS-related investigations and treatment are free of charge to the patient according to the Tanzanian National Policy on HIV/AIDS care and treatment. The villages of residence of the HIV+ patients were used to sample match HIV– individuals. These villages were located in the Mbulu, Hanang and Babati districts of the Manyara region and Iramba district of the Singida region (Fig. 1). All study districts were characterized by poor infrastructure and living conditions, lack of sanitation, and farming as the main source of income. In 2008, the Manyara region reported that 19.6% of rural households reared pigs with the largest number of pigs found in the Mbulu district (51 198 pigs, 53.1% of the pig population in the Manyara region), followed by the Babati district (19 587 pigs, 20.3%) and the Hanang district (15 310 pigs, 5.9%) [36]. The Iramba district reported 42 073 pigs, which corresponds to 86% of the total pig population in the Singida region [37].

According to the Tanzania HIV/AIDS and Malaria Indicator Survey (THMIS) 2011-12 [38], the overall HIV prevalence in Tanzania was estimated to be 5.1% among adults aged 15–49 years (6% among women, 4% among men) during our study period. In the same report, prevalence proportions of 1.5% for the Manyara region and 3.3% for the Singida region were estimated. It is believed that HIV emerged in these regions relatively late (mid 1990s), but has steadily increased ever since and already reached the country’s average in some districts like Babati [39].

### Study design and study population

**Fig. 2:**
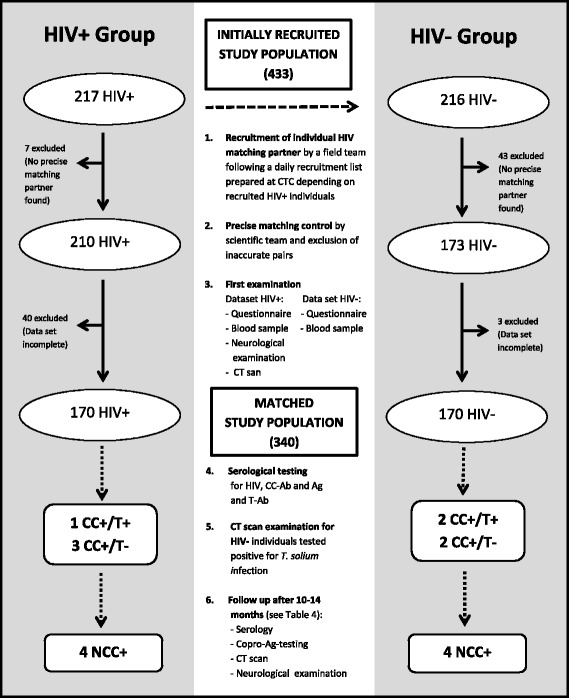
Flow chart of the initially recruited and matched study populations. HIV: human immunodeficiency virus, CT: computed tomography, CC: cysticercosis, T: taeniosis, NCC: neurocysticercosis, Ab: antibody; Ag: antigen

A cross-sectional study was conducted among 170 HIV+ and 170 age (+/− 10 years), gender and village of residence-matched HIV– individuals. The rationale for matching was to limit variation in exposure to *T. solium* eggs among HIV+ and HIV– participants. Participants were recruited from January 2011 to February 2013. Every day, HIV+ patients attending CTC for regular clinical check-ups and/or HAART were invited to participate in the study. Age, gender and village of residence of each consenting HIV+ patient were entered into an Excel sheet on a daily basis. For the age range of the individual matching partner a range of +/− 10% (with minimum and maximum target age) was calculated. Following this list, a field team including one nurse from CTC and one research assistant went each day to the appropriate villages to identify matching HIV– participants. With the help of village headmen/headwomen and local helpers, potential candidates were visited at their homes and asked to voluntarily participate in this study. The overall recruitment was stopped when a total of 433 participants, resulting in 170 matching pairs, had been identified (Fig. 2). Initial HIV testing of individuals enrolled in CTC was performed using two rapid Ab-tests according to the Tanzanian national algorithm for HIV testing: Alere Determine HIV-1/2 (Abbott laboratories, Abbott Park, IL, USA) and Uni-Gold™ Recombigen® HIV-1/2 (Trinity Biotech, USA). In the case of a positive reading in the Alere Determine HIV-1/2 test the serum sample was subjected to the second rapid Ab-test: Uni-Gold™ Recombigen® HIV-1/2 test. Concordant positive results were interpreted as positive for HIV-Ab. Discordant results were interpreted as inconclusive and the sample was sent to the regional hospital for confirmatory test using two ELISA tests: Enzygnost anti-HIV 1 + 2 Plus ELISA (Behring, Marburg, Germany) and Well-coenzyme HIV recombinant ELISA (Murex, Dartford, England). In this study, HIV– individuals were defined as participants who tested negative using the Alere Determine HIV-1/2 test and HIV+ individuals as participants tested positive following the algorithm described above. Clinical staging of HIV/AIDS was performed according to the revised World Health Organization (WHO) guidelines and HAART treatment status of all HIV+ was noted [40]. Most HIV+ participants (164 or 96%) were receiving HAART and none were under epilepsy or anti-inflammatory treatment at the time of recruitment. For ethical reasons, only individuals aged at least 9 years old were included in the study. Pregnant women were excluded from the study.

Consenting individuals were invited to answer a questionnaire, to undertake a clinical examination and to provide a blood sample for serological testing for taeniosis and CC. Neuroimaging was offered to all HIV+ participants and to only those HIV– participants positive to TSOL serology for ethical reasons.

### Blood sample collection and laboratory testing

After counselling by a specially trained nurse, 10 ml of whole blood was collected by venipuncture from each participant. HIV+ and HIV– individuals were informed about the HIV test results immediately thereafter. CD4^+^ counts were determined for all HIV+ patients using a fluorescent activated cell sorter machine (FACScount™, BD Biosciences, US) at the central laboratory of HLH. Blood samples from HIV– individuals were transported in cool boxes to the laboratory and immediately placed into refrigerators until required for further processing. Blood from HIV+ individuals was refrigerated in the HLH central laboratory immediately after drawing. After 1 to 6 h, serum was separated and stored at −20 °C until serological testing for TSOL was performed. On average, two vials with 2 ml of serum were obtained from each individual. Four to twelve months later, samples were shipped to the CDC in Atlanta, USA, for further analyses and one safety back up sample remained at the Parasitology Laboratory of MUHAS to avoid total loss in case of any damage to the sample during the long shipment. Two tests were used to detect the presence of Ab to CC (LLGP-EITB and rT24H-immunoblot), one test was used to detect the presence of antigen (Ag) to CC (Ag-ELISA) and one test was used to detect the presence of Ab to taeniosis (rES33-immunoblot). LLGP-EITB is an enzyme-linked immunoelectrotransfer blot that detects CC-specific Ab to any of seven glycoproteins [41, 42]. The rT24H-test is an immunoblot method that detects CC-specific Ab to a *T. solium* recombinant Ag [43]. The Ag-ELISA that detects *Taenia*-Ag in serum is a monoclonal Ab (B158/B60 Ab) based capture ELISA and was performed following the modified protocol described by Dorny et al. [44]. The rES33-test is an immunoblot method that detects adult *T. solium* specific Ab using a recombinant protein derived from the excretory-secretory proteins of the adult tapeworms [45, 46]. Ab to both of the recombinant peptides, rT24H and rES33, were assessed in the same test. All test results were interpreted independently and blinded by two scientists with many years of experience in reading these *T. solium* in-house tests. For quality assurance each positive test was repeated at least once as well as 15% randomly selected negative samples.

### Definitions

*T. solium* positive individuals (TSOL+) were defined as those with a positive result in at least one of the four serological tests described above while those negative in all tests were considered *T. solium* negative (TSOL–). Positive CC cases were defined as those individuals positive to the LLGP-EITB, rT24H-immunoblot or Ag-ELISA test. Taeniosis cases were defined as individuals positive to the rES33-immunoblot test. NCC cases were defined following the revised criteria of Del Brutto [10]. Viable cysts (active NCC) were defined as cystic lesions (with or without visible scolex) or lesions with ring enhancement. Calcified cysts (inactive NCC) were defined as hyperdense lesions with no sign of ring enhancement [47].

### Neuroimaging

All HIV+ individuals underwent CT examination at baseline in a Toshiba Auklet Single Slice Spiral CT machine at HLH. HIV–/TSOL+ participants received their CT examination at the Aga Khan Health Centre in Arusha since the CT machine at HLH did not work at the time of the follow-up examinations. All follow-up CT scans of HIV+/TSOL+ were also performed in Arusha for the same reason. This health center used a Siemens Somatom Emotion CT machine. Both machines provided 5 mm slices and took 64 slices per picture. Intravenous contrast medium was used in all patients. CT scan images were evaluated by the local radiologists and sent to the Department of Neurology at the Technical University Munich (TUM) for a second blinded evaluation by a neurologist specialized in NCC diagnoses.

### Research questionnaire

Each participant was asked to answer a socio-demographic and a medical history interview questionnaire. Interviews with HIV+ individuals were performed by a clinical officer at the CTC clinic and with HIV– individuals by a trained nurse in their villages of residence. The study nurses delivered the questionnaires at the CTC clinic. The questionnaire was adapted from a previous validated questionnaire used by the *Cysticercosis Working Group in Eastern and Southern Africa (CWGESA)* and addressed socio-demography, pork consumption habits as well as hygiene and sanitary practices [48]. Questions with an emphasis on self-reported past and present neurological disorders, such as acute and chronic headaches, peripheral nervous system (PNS) and CNS signs and symptoms including epileptic seizures as well as psychiatric disorders were included; so were questions on family history of neurological disease. HIV+ individuals were also asked about their past HIV history including opportunistic infections, HAART duration as well as compliance, amongst others. The questionnaire was developed in English, translated into Swahili and back-translated to English by two independent people. A standard neurological examination was performed on all HIV+ individuals by a medical doctor specializing in neurology.

### Follow-up of seropositive individuals

Ten to fourteen months elapsed between sampling and receipt of the serological tests results due to logistical reasons and challenges in tracing patients, therefore HIV+/TSOL+ participants were invited for a second CT scan and blood collection (10 ml from each individual) so that appropriate treatment could be provided. This second clinical examination and follow-up CT scan were performed shortly before treatment and blood samples were taken at HLH. Participants identified HIV–/TSOL+ were invited for their first CT scan and neurological examination, and second blood drawing before treatment. Taeniosis positive participants were treated with niclosamide and symptomatic NCC cases with albendazole and dexamethasone. Present CD4^+^ counts of HIV+ individuals were obtained and all serum samples were again shipped to the CDC in Atlanta for testing as described above. In addition, before treatment, TSOL+ participants were asked to provide three stool samples for copro-Ag-ELISA testing at the CDC, following a protocol described by Guezala et al. [49]. Due to financial limitations in our study copro-Ag testing could only be performed during follow-up.

### Statistical analysis

Analysis of associations between potential risk factors including HIV (independent variables) and TSOL (dependent variable) was performed by bivariate approximative tests (*χ* ^2^-tests) and exact tests (Fisher’s tests) using Epi Info, version 3.3.2 (CDC, Atlanta, GA). Significant differences were defined as *p-*values below 0.05. McNemar tests were used for the paired analysis associations between the HIV status and TSOL, as well as for determining all the neurological outcomes. Matched prevalence proportion ratios (mPPR) and their 95%*CI* were obtained using the csmatch command in Stata 14.0. Statistical significance of associations between HIV and other factors among all matched and unmatched recruited participants and among the HIV positive group were evaluated using the chi-square test for categorical variables and the Kruskal Wallis tests for continuous variables. Matched analyses were performed using Stata 14.0.

## Results

### Demographic characteristics of HIV+ and HIV– individuals of the matched study population

Initially, 217 HIV+ individuals at CTC and 216 HIV– individuals were recruited to participate in the study. The initial aim of this recruitment was to identify a minimum of 150 matched pairs of HIV+ and HIV– individuals. After the initial recruitment, 40 HIV+ and three HIV– individuals were excluded due to a loss of follow-up or because the individual refused to participate further in the study. Full data sets comprised a completed questionnaire, serological results as well as at least one cranial CT examination and, for HIV+ participants, a neurological examination. After applying the matching criteria, seven additional HIV+ individuals and 43 HIV– individuals were excluded from the group of 177 HIV+ and 213 HIV– individuals, resulting in 170 matching HIV+/HIV– pairs available for analysis (Fig. 2).

The socio-demographic characteristics of the matched groups are reported in Table 1. As determined by the design, each group was 50% male and the median age was 39 years and 40 years in the HIV+ and HIV– matched participants groups, respectively. The most frequent tribe was Iraqw (42.6%), followed by Nyiramba (35.6%), Nyisanzu (8.5%) and other tribes (13.2%). Most of study participants lived in the Iramba district (45.6%), followed by the Mbulu (38.5%), Hanang (10.9%) and Babati districts (5.0%) (Table 1).

**Table 1:** Demographic characteristics of HIV+ and HIV– individuals of the matched study population

Variables	HIV+ (170)		HIV– (170)		Total (340)		*p-*value
	*n*	%	*n*	%	*n*	%	
Gender							1.00^a^
Male	85	50.0	85	50.0	170	50.0	–
Age group							0.50*
9–19 years	5	2.9	6	3.5	11	3.2	0.61*
20–39 years	70	41.2	82	48.2	152	44.7	0.23*
40–59 years	83	48.8	72	42.4	155	45.6	0.12*
60–74 years	12	7.1	10	5.9	22	6.5	0.88*
District							0.96*
Mbulu	62	36.5	69	40.6	131	38.5	0.44*
Iramba	82	48.2	73	42.9	155	45.6	0.33*
Hanang	18	10.6	19	11.2	37	10.9	0.86*
Babati	8	4.7	9	5.3	17	5.0	0.80*
Tribe							0.30
Iraqw	66	38.8	79	46.5	145	42.6	0.15
Nyiramba	64	37.6	57	33.5	121	35.6	0.43
Nyisanzu	13	7.6	16	9.4	29	8.5	0.56
Other tribes	27	15.9	18	10.6	45	13.2	0.15
Marital status							0.10
Married	130	76.5	143	84.1	273	80.3	0.08
Single	37	21.8	24	14.1	61	17.9	0.07
Divorced	3	1.8	3	1.8	6	1.8	1.00
Education							0.01
School not attended	37	21.8	18	10.6	55	16.2	–
School attended	133	78.2	152	89.4	285	83.8	–
Occupation							0.02
Peasant	149	87.6	155	91.2	304	89.4	0.53
Housewife	6	3.5	0	0.0	6	1.8	0.01
Business man/woman	3	1.8	0	0.0	3	0.9	0.08
Medical occupation	1	0.6	5	2.9	6	1.8	0.10
Student	6	3.5	1	0.6	7	2.1	0.06
Other	5	2.9	9	5.3	14	4.1	0.28

*HIV* human immunodeficiency virus

*The factors gender, age group, and district were considered for matching between HIV+ and HIV–

### HIV related characteristics

At the time of recruitment 161 of 170 HIV+ individuals were on regular HAART therapy, three had discontinued treatment during recent years at least once, and six had not yet started HAART. The most commonly used HAART drugs were the first-line combinations lamivudine/zidovudine or lamivudine/stavudine/nevirapine (76.5%). 46 (27.1%) HIV+ patients reported opportunistic infections in the past year, mostly pulmonary tuberculosis and other respiratory diseases as well as herpes zoster and other infectious skin diseases. The distribution of HIV/AIDS stages at the time of recruitment was: stage I: 71.8%, stage II: 9.4%, stage III: 16.5%, and stage IV: 2.4%.

### Association between HIV and relevant past medical/neurological history, current neurological deficits, and TSOL risk factors among the matched pairs

HIV+ patients had significantly more history of acute headaches (mPPR: 2.38; 95%*CI*: 1.44–3.93) and a past history of psychiatric disorders (mPPR: 2.50; 95%*CI*: 1.02–6.15) than their HIV– counterparts. HIV+ individuals also tended to have more current overall neurological deficits (mPPR: 7.00; 95%*CI*: 0.86–56.89) and, specifically, peripheral neurological deficits (like anesthesia and hypoesthesia of legs and arms) (mPPR: 2.60; 95%*CI*: 0.93–7.29), but the difference was not significant (Tables 2 and 3).

**Table 2:** Matched prevalence proportion ratios (mPPR) and their 95%*CI* for risk factors of TSOL, relevant past medical/neurological history and current neurological deficits

Factor (total number of pairs with data available)	Number of pairs				mPPR (95%*CI*)
	HIV+ w f/HIV– w f	HIV+ w f/HIV– wo f	HIV+ wo f/HIV– w f	HIV+ wo f/HIV– wo f	
History of overall headaches (162)	8	36	12	114	2.20 (1.39–3.48)
History of acute headaches (170)	7	31	9	123	2.38 (1.44–3.93)
History of chronic headaches (170)	0	6	4	160	1.50 (0.42–5.32)
Past or present epileptic seizures (170)	0	2	1	167	2.00 (0.18–20.06)
Current CNS symptoms (170)	0	7	1	162	7.00 (0.86–56.89)
Current PNS symptoms (164)	0	7	5	152	2.60 (0.93–7.29)
Any positive test for TSOL (170)	0	4	4	162	1.00 (0.25–4.00)
Positive for CC-Ag	0	1	0	169	Undefined
Positive for CC-Ab (170)	0	4	4	162	1.00 (0.25–4.00)
Positive for taeniosis-Ab (168)	0	3	2	163	0.50 (0.05–5.51)
Consumes pork (164)	95	18	38	13	0.85 (0.75–0.96)
Consumes undercooked pork (170)	0	9	10	151	0.90 (0.37–2.21)
Consumes pork at least once a month (170)	3	19	20	128	0.76 (0.42–1.38)
Has seen cysts in pigs (170)	0	3	1	166	3.00 (0.31–28.84)
Has access to a latrine (170)	163	2	5	0	0.98 (0.95–1.01)
History of tapeworm carrier in family (170)	1	6	2	161	2.33 (0.70–7.82)
Handwashing before eating (170)	0	11	20	139	0.55 (0.26–1.15)
Anthelminthic treatment in the past year (170)	3	10	20	137	0.57 (0.30–1.05)

*mPPR* matched prevalence proportion ratio, *CI* confidence interval, *HIV* human immunodeficiency virus, *w* with, *wo* without, *f* factor, *CNS* central nervous system, *PNS* peripheral nerval system, *TSOL T. solium* taeniosis/cysticercosis, *CC* cysticercosis, *Ag* antigen

**Table 3:** Past neurological history, public health and diagnostic data of HIV+ and HIV– individuals of the matched study population

Variables	HIV+ (170)		HIV– (170)		Total (340)		*p*-value
	*n*	%	*n*	%	*n*	%	
History of acute headaches							
Yes	38	22.4	16	9.4	54	15.9	*<0.01*
History of chronic headaches							
Yes	6	3.5	4	2.4	10	2.9	0.52
Past or present epileptic seizures							
Yes	4	2.4	3	1.8	7	2.1	0.70
History of psychiatric disorders							
Yes	15	8.8	6	3.5	21	6.2	*0.04*
Current CNS symptoms							
Yes	7	4.1	1	0.6	8	2.4	*0.03*
Current PNS symptoms							
Yes	13	7.6	5	2.9	18	5.3	0.05
CC-Ag							
Positive	1	0.6	0	0.0	1	0.3	0.32
CC-Ab by LLGP-EITB							
Positive	4	2.4	4	2.4	8	2.4	1.00
CC-Ab by rT24H-blot							
Positive	4	2.4	4	2.4	8	2.4	1.00
Taeniosis-Ab							
Positive	1	0.6	2	1.2	3	0.9	0.56
NCC^a^							
Positive	4	2.4	4	2.4	8	2.4	1.00
History of tapeworm carrier in family							
Yes	7	4.1	3	1.8	10	2.9	0.20
Handwashing before eating							
Yes	12	7.1	20	11.8	32	9.4	0.14
Anthelmintic treatment in the past year							
Yes	12	7.1	25	14.7	37	10.9	*0.02*

*CNS* central nervous system, *PNS* peripheral nervous system, *HAART* highly active antiretroviral therapy, *CC* cysticercosis, *Ag* antigen, *Ab* antibody, *LLGP-EITB* lentin-lectin glycoprotein electroimmunosorbent blot, *NCC* neurocysticercosis

^a^According to the revised diagnostic criteria proposed by Del Brutto [10]

Among the 164 pairs with this information available, HIV+ participants reported consuming pork significantly less often than HIV– participants (mPPR: 0.85; 95%*CI*: 0.75–0.96), but most of those consuming pork reported to eat pork less than once a month and only properly cooked. Only 1.2% of all study participants reported ever seeing cysts in pork. Of all matched study participants, 97.9% reported having access to latrines at home, and 98.2% of those reported always using the latrine. Only 9.4% of the participants reported washing hands before food consumption; the HIV+ participants tended to wash their hands less often than the matched HIV– participants (mPPR: 0.55; 95%*CI*: 0.26–1.15) (Tables 2 and 3).

There were six pairs where the HIV+ individual reported living in a household with a member with a past history of tapeworm infection, while the HIV– matched participant did not; in only two pairs did the HIV– participant reported such history and the HIV+ did not (mPPR: 2.33; 95%*CI*: 0.70–7.82). Fewer HIV+ participants tended to report anthelminthic treatment in the past than the matched HIV– participants (mPPR: 0.57; 95%*CI*: 0.30–1.05), but the difference was not significant (Tables 2 and 3).

### Distribution of TSOL tests results among the matched pairs and in the initially recruited population

There was no association between HIV and TSOL in this population with four discordant pairs each of HIV+/TSOL+ with HIV–/TSOL– and HIV+/TSOL– with HIV–/TSOL+ (mPPR: 1.0; 95%*CI*: 0.25–4.00). This corresponds to an overall prevalence of 2.4% (95%*CI*: 0.6–5.9) of TSOL among both HIV+ and HIV– participants. Comparing detailed test results, no significant differences were observed between matched HIV+ and HIV– individuals regarding the sero-prevalence of taeniosis-Ab (mPPR: 0.5; 95%*CI*: 0.05–5.51), CC-Ab (mPPR: 1.0; 95%*CI*: 0.25–4.00), and CC-Ag (mPPR undefined because there was only one discordant pair with HIV+ positive to the Ag-ELISA and HIV– negative to Ag-ELISA) were detected (Fig. 2, Tables 2 and 3).

Of the 404 participants initially recruited with sera available, which include the 340 matched participants, the prevalence of CC-Ab was 2.5% (95%*CI*: 1.2–4.5). For more clinical, serological and risk factor data of those 10 TSOL+ individuals refer to Additional file 2: Table S1. The prevalence of Ab to taeniosis was 1.2% (95%*CI*: 0.4–2.9). All five of 404 rES33 positive individuals also had Ab to CC-Ag or positive Ag-results. Only three (0.7%; 95%*CI*: 0.4–2.9) participants were positive for the presence of CC-Ag. The prevalence of TSOL was slightly higher among those presenting with headaches or seizures (3.7%; 95%*CI*: 0.8–10.4) than among those without such symptoms (2.2%, 95%*CI*: 0.9–4.4), but this difference was not statistically significant (Tables 2 and 3).

The district of origin and serological results were available for 399 out of 433 initially recruited individuals. The prevalence proportion of TSOL was considerably higher among participants living in the Mbulu district (5.2%, 95%*CI*: 1.9–10.4) than in other districts (0.8%; 95%*CI*: 0.1–2.8) (Fig. 1), a difference which was statistically significant (*p* = 0.008) (Table 1).

### Association between CD4^+^ counts, HIV stages, HAART and TSOL

There were only four HIV+ participants positive for any TSOL test. Median CD4^+^ counts of the four HIV+/TSOL+ individuals were 358 cells/μl (range 170 to 554) while it was 399 cells/μl (range 9 to 1 878) among the 166 HIV+ participants of the matched population negative to all TSOL tests (*p* = 0.88). Of four HIV+/TSOL+, three individuals were classified in HIV stage I and one in stage III, a distribution similar to that found in those with all TSOL tests negative (*p* = 0.88). All four HIV+/TSOL+ individuals had been receiving HAART for 4 years or less (Table 4).

**Table 4:** Radiological and serological results of TSOL+ individuals of the matched and initially recruited population

	P 1	P 2	P 3	P 4	P 5	P 6	P 7	P 8	P 9	P 10
Matched study group	yes	yes	yes	yes	yes	yes	yes	yes	no	no
Age	50	46	45	60	46	28	27	51	38	14
Gender	f	m	m	m	m	f	f	m	m	m
First clinical examination										
HIV parameters										
HIV status	+	+	+	+	-	-	-	-	-	-
HIV stage	I	I	I	III	-	-	-	-	-	-
HAART drugs	ZDV/3TC/EFZ	ZDV/3TC/EFZ	ZDV/3TC/EFZ	ZDV/3TC/EFZ	-	-	-	-	-	-
HAART treatment since (years)	<1	3	3	4	n.a.	n.a.	n.a.	n.a.	n.a.	n.a.
NCC symptoms/signs	-	-	-	headaches	-	severe headaches	-	-	epi	epi, enc^a^
CT diagnosis										
Calcified cysts	+	-	+	+	o	o	o	o	+	-
Viable cysts	-	-	-	-	o	o	o	o	-	+
Other NCC relevant findings	-	-	-	-	o	o	o	o	-	hydrocephalus
Laboratory diagnosis										
CD4^+^ counts	554	433	276	170	n.a.	n.a.	n.a.	n.a.	n.a.	n.a.
CC- Ab	+	+	+	+	+	+	+	+	+	+
CC-Ag	-	-	-	+	-	-	-	-	-	+
Taeniosis-Ab	+	-	-	-	-	-	+	+	+	+
Second clinical examination										
NCC symptoms/signs	x	x	-	-	-	severe headaches	-	x	d	epi, enc^a^
CT diagnosis								x		
Calcified cysts	x	x	+	+	+	-	+	x	d	+
Viable cysts	x	x	-	-	-	-	-	x	d	+
Other NCC relevant findings	x	x	-	-	-	hydrocephalus^b^	-	x	d	hydrocephalus
Laboratory findings										
CD4^+^ counts	521	400	368	184	n.a.	n.a.	n.a.	n.a.	d	n.a.
CC-Ab	+	+	-	-	+	x	x	x	d	x
CC-Ag	-	-	-	+	-	x	x	x	d	x
Taeniosis-Ab	+	-	-	-	-	x	x	x	d	x
Taeniosis-copro-Ag	-	-	-	-	-	-	-	x	d	x

*P* patient number, *m* male, *f* female, *HIV* human immunodeficiency virus, *HAART* highly active antiretroviral therapy, *ZDV* zidovudine, *3TC* lamivudine, *EFZ* efavirenz, *NCC* neurocysticercosis, *epi* epilepsy, *enc* encephalopathy, *CT* computed tomography, *CD4* ^*+*^ CD4^+^ T-lymphocyte cell counts, *n.a.* not applicable, because in the group of HIV– these parameters were not tested and no HAART was taken, *CC* cysticercosis, *Ab* antibodies, *Ag* antigen, + positive, − negative, *o* was not taken at once due to ethical concerns of performing a CT scan in healthy individuals, but was offered when the positive test serology was confirmed, *x* patient refused or was not found, *d* patient died before 2^nd^ examination with unclear diagnosis (strong headaches and abdominal pain reported before death)

^a^Patient was unconscious with signs of brainstem involvement and generalized increased muscle tone leading to flexion contractures of all four limbs, most likely in the context of increased intracranial pressure

^b^Mass in 4^th^ ventricle causing obstructive hydrocephalus

### Radiological and serological results of TSOL+ individuals among the matched pairs and initially recruited population

Details on the four HIV+ patients (P1-P4) and the four HIV– patients (P5-P8) who were also TSOL+ in addition to two HIV– participants (P9-P10) who could not be matched but were TSOL+ (initially recruited study population; Fig. 2) are provided in Table 4. More clinical details on these ten patients are shown in Additional file 2: Table S1.

Overall, all four HIV+/TSOL+ and three of four HIV–/TSOL+ participants of the matched study population agreed to have at least one CT scan at the time of their first and second clinical examination. The two unmatched HIV–/TSOL+ individuals (P9, P10) received a CT scan at their first clinical examination due to the severe clinical condition they were in (epilepsy, encephalopathy). None of the 166 HIV+/TSOL– participants presented with brain lesions compatible with NCC, but three (P1, P3 and P4) of the four HIV+/TSOL+ had definitive NCC. The fourth participant did not show any lesions in the brain (P2). This would suggest an overall specificity of 99.4% (95%*CI*: 96.7–100) of the CC tests (Ag-ELISA, LLGP-EITB, rT24H-immunoblot) to detect NCC among the HIV+ patients in this population. There were too few cases to estimate the sensitivity with any precision. Overall, there was perfect agreement between the EITB and rT24H for the detection of Ab to CC (Tables 3 and 4).

Among the three matched HIV–/TSOL+ participants with CT scan results, two were found to have definitive NCC (P5, P7) and one other had probable NCC (P6). The latter presented with an obstructive hydrocephalus and a mass in the 4^th^ ventricle. Among all HIV– participants positive to TSOL and receiving neuroimaging, four out of five had lesions of NCC, suggesting that, although there were only small numbers, HAART may not impact on the proportion of NCC among those positive for CC in people with HIV/AIDS. The proportion of NCC was therefore 1.8% among the matched HIV+ participants, but cannot be estimated in the matched HIV– participants since only three had a CT scan.

Among the seven NCC cases, five presented with calcified lesions without perilesional edema and were free of any viable cysts. Among the two unmatched HIV–/NCC participants, one was found to have multiple viable cysts in the parenchyma, some of them with perilesional edema (P10), and the other had several calcified cysts (P9). The number of detected lesions per NCC case in this study ranged from one to ten, except in P10, where more than 20 active lesions could be identified. No lesions outside the parenchyma were detected (Table 4).

### Description of symptoms among patients with NCC

Four (1 HIV+, 3 HIV–) of eight participants with definitive or probable NCC presented with neurological symptoms. Two of the symptomatic participants presented with headaches (1 HIV+ with acute and 1 HIV– with chronic headaches) and two HIV– individuals with epilepsy. One HIV– participant (P10), a boy aged 14 years, was unconscious at the time of admission to HLH with signs of brainstem involvement and generalized increased muscle tone leading to flexion contractures of all four limbs, most likely in the context of increased intracranial pressure. P10 was the only participant with positive serological results for all tests (CC-Ab, CC-Ag and taeniosis-Ab). The CD4^+^ counts of the only symptomatic (presenting with acute headaches) HIV+ individual (P4) was 170 cells/μl, the lowest level of the four HIV+ individuals who were positive to TSOL (Table 4).

### Follow-up of TSOL+ individuals

Of the ten participants found positive to TSOL at baseline, three were lost to follow-up, including one unmatched HIV– participant who died for unclear reasons (strong headaches and abdominal pain reported before death). Five (4 HIV+ and 1 HIV–) of the seven followed participants provided sera and six (4 HIV+ and 2 HIV–) provided a stool sample. Seven (4 HIV+ and 3 HIV–) participants had a stool examination at the time of follow up, but all of them had a negative copro-Ag-ELISA test result. The only HIV+ participant positive to rES33 at baseline was still positive at follow-up. Among the five participants positive to CC at baseline, one HIV+ participant became sero-negative to the tests detecting Ab. Interestingly, this participant (P3) had an increase of 92 cells/μl in CD4^+^ (from 276 to 368 cells/μl; increase of 33% from baseline), in contrast to the three other HIV+ participants for whom the CD4^+^ counts decreased slightly or remained similar. Only three participants accepted to undergo a second CT examination. Two of them did not show any changes, whereas P10, who had shown several viable cysts on the first CT scan, revealed mostly calcifications as well as a few viable cysts. This young (14-year-old) patient was hospitalized and, after serological and radiological confirmation, received anti-inflammatory (dexamethasone) and anthelminthic (albendazole, niclosamide) treatments repeatedly during the whole study period. There were no major improvements in the symptoms after treatment and the patient was transferred to a home for children with special needs after the study. Overall, there was no change in the clinical presentation of all the TSOL+ individuals at follow-up. None of the asymptomatic NCC cases reported new clinical signs/symptoms (Table 4, Additional file 2: Table S1).

## Discussion

TSOL has only been investigated patchily with contradictory results in people living with HIV/AIDS and the impact of CD4^+^ counts, HAART duration and HIV stages on the incidence of *T. solium* and the exacerbation of NCC have not been explored so far. Depending on the endemicity of TSOL, between 10 and 20% of HIV+ individuals may also present with TSOL and/or suffer from NCC, but there are currently no recommendations for adequate case management in cases of co-infection [31]. This important gap for controlling TSOL/NCC has largely been overlooked. Our study matched HIV+ to HIV– participants by age, gender and village of residence to adjust for potential environmental contamination with eggs of *T. solium* and the varying immunological response in relation to age and gender. To our knowledge, our study is the first to adjust for such factors in people living with HIV/AIDS and was designed to address these knowledge gaps. Unfortunately, the prevalence of taeniosis, CC and NCC was a lot lower than had been reported in the region by past studies [9, 17], and limited our ability to fully explore these questions with confidence.

We found a prevalence proportion of Ab to CC of 2.4% among the matched participants and of 2.5% in the initially recruited population, with no major differences between the HIV+ and HIV– participants. These prevalence rates of Ab to CC were similar to a 2.2% prevalence rate reported among healthy individuals in Kenya [50]. However, our estimate is much lower than that found in a study conducted at the same time in communities of the Mbulu district where a prevalence proportion of 16.3% was reported when using a commercially available Western blot test (LDBIO Diagnostics) [51]. When using data from all participants initially recruited, the prevalence of TSOL was highest in the Mbulu district (5.2%), and this was true among HIV+ and HIV– participants, but was still lower than that found by Mwang’onde et al. [51]. Possible explanation for these differences are the use of a different diagnostic test for the detection of Ab, possible selection bias of participants as no details on sampling is provided by the authors of a previous study [51], and possible clustering of CC in certain villages within the Mbulu district which was already described by Ngowi at al. [52]. Clustering of CC by villages in Africa has also been observed in pigs in the Mbulu district and in humans living in other countries [16, 52].

The prevalence of CC-Ag positive individuals in our study is also considerably lower than that found in other countries of southeastern Africa: Zambia (5.8%), Tanzania (16.7%), and the Democratic Republic of Congo (21.6%) [15–17, 53], and was lower than the overall prevalence of 7.3% for Africa presented in [54]. However, a study from Burkina Faso recorded varying proportions, from 0.0 to 11.5% in 60 villages, which partially supports our findings [55].

In our study, NCC was confirmed by serology and CT scan in about 2% of HIV+. In a study conducted in India, of 100 HIV+ serum samples two (2%) were detected with CC-Ab by EITB [56]. In Mexico, only 1.1% of NCC have been reported in a study among HIV/AIDS cases, compared with 2.4% in control autopsies [33]. Furthermore, a study conducted in Mozambique found a CC-Ab proportion of 10.2% in 601 HIV+ individuals by a commercial multiplex Western Blot IgG test for several parasites (LDBIO Diagnostics) [31].

Overall, it has to be considered that due to the matched design in this study and no use of a cross-sectional approach, obtained prevalence estimates have to be interpreted carefully. No cluster analysis could be performed for this reason too.

Four TSOL individuals, two matched individuals (1 HIV+, 1 HIV–) as well as two initially recruited individuals (2 HIV–) presented with neurological symptoms. Our results are limited by the fact that symptomatic NCC cases with a negative CC-Ab and CC-Ag titer and people with extraparenchymal or spinal lesions might have been missed. Ethical restrictions did not allow for further radiological follow-up of sero-negative HIV– participants and funds as well as logistics made the performance of magnetic resonance imaging (MRI) impossible.

Of all eight NCC cases identified in our wider study population four were classified as asymptomatic and four as symptomatic NCC cases. This correlates with findings of other studies that proposed that around 50% of NCC cases remain asymptomatic [4, 56]. The two reported neurological symptoms/signs were headaches and epilepsy, which is consistent with the main neurological presentations of NCC [2–4]. All asymptomatic NCC cases showed one to five intraparenchymal calcified lesions in the CT scan, and none of those followed up developed neurological symptoms 8 to 10 months later. This does not rule out that these individuals may become symptomatic in the future.

The prevalence of taeniosis-Ab in the initially recruited population was 1.2%, the first such estimate for northern Tanzania. However, this population is not representative of the community but was selected to match residence of HIV+ individuals receiving care at the HLH. Data on taeniosis-Ab are still scarce worldwide, mostly due to the unavailability of a commercial diagnostic test [57]. The rES33-immunoblot in-house assay has been reported to have a sensitivity of 98% and a specificity of 99% for the detection of the adult stage of *T. solium* in human intestines [45]. However, the presence of taeniosis-Ab only reflects exposure to the adult tapeworm and cannot differentiate past from present infections. Previous estimates of the prevalence of adult tapeworm infections measured with a copro-Ag-ELISA in endemic areas vary from 0.1 to 4.0% in community-based settings, with only one study from Zambia reporting a prevalence of 11.9% [13, 15–18]. In our study, all individuals with a positive taeniosis-Ab titer lived in the Mbulu district (Fig. 1). In addition to the possible natural clustering of TSOL, in contrast to the other districts, Mbulu had only recently started annual mass drug administration (MDA) of anthelminthic drugs which may have reduced the natural taeniosis prevalence.

Our analysis of factors for TSOL infection revealed that the HIV+ group reported washing their hands less often, was more exposed to people with taeniosis in their household but had taken fewer anthelminthic drugs in the past (as specifically prescribed treatment or in mass drug administration initiatives). However, they also consumed less pork (Tables 2 and 3), so there could be a slight tendency that the HIV+ group may be slightly greater at risk of CC and a little less to taeniosis, based on known infection routes [2–4]. However, serological findings of our study could not support this hypothesis as in our study four HIV– individuals had a positive taeniosis-Ab titer in comparison to only one HIV+ individual.

In the present study, we could not detect any difference in the prevalence of taeniosis, CC and NCC between matched HIV+ and HIV– individuals. Our results are in accordance with that of Walson et al., who suggested that helminth prevalence in HIV+ individuals is similar to the general population prevalence in endemic settings [58], although others have found intestinal helminths - like *Strongyloides stercorali*s - to be more prevalent in HIV+ people [59, 60]. However, a clear drawback of our study was the seemingly low prevalence of TSOL in our overall study population and therefore differences between the HIV+ and HIV– individuals may have gone unnoticed. Furthermore, there was no difference in the presence of CC-Ab between matched HIV+ and HIV– individuals. A reduced production of Ab against protozoans such as *Toxoplasma gondii* has been discussed in HIV+ individuals, as well as the possible failure of serological diagnostic tests to detect these co-infections in severely immunocompromised individuals [61]. In our study, CC-Ab positive HIV+ individuals had moderately reduced to normal CD4^+^ counts and were probably still immunocompetent enough to produce Ab. CC in HIV+ individuals, including Ab and/or Ag-positive individuals, was distributed equally (1/4) in the four groups of individuals with defined CD4^+^ counts (group I: ≤200; group II: 201-350; group III: 351-500; group III: >500). Therefore, CC did not seem to be associated with CD4^+^ counts in our study.

The single HIV+/NCC case with neurological symptoms presented with acute headaches, calcified cysts, CD4^+^ counts of 170 cells/μl and was HIV stage III. All asymptomatic HIV+/NCC cases were HIV stage III. This result is in agreement with previous studies reporting symptomatic HIV+/NCC cases in individuals with CD4^+^ counts <250 cells/μl [27, 32]. However, acute headaches in this case might also be linked to HIV infection, HAART, or reasons other than NCC.

Our results showing the absence of viable cysts or perilesional edemas lesions among HIV+/NCC cases are in contrast with a review of 27 HIV+/NCC cases with multiple parenchymal active lesions (viable cysts and enhancing lesions) as the most frequent radiological findings [32]. Beside genetic aspects, of the host and/or the parasite, and lower infection pressure the prevailing of cerebral calcifications in our HIV+ study population on the one hand may point towards a still functional immune system that is able to eliminate the parasite and on the other hand may be due to low infection pressure. Another reason might be that the HIV+/NCC cases acquired HIV after contracting TSOL.

Although this study was designed to allow similar levels of exposure to *T. solium* eggs in the HIV+ and HIV– groups, it had some important limitations which should be noted. First, the very small number of TSOL+ individuals in both the HIV+ and HIV– groups meant that we had very limited statistical power to identify any differences. Such result was surprising given that endemic levels of CC/NCC were recently reported in the same study area [9, 51].

Second, the vast majority of the HIV+ participants were receiving HAART, meaning that the impact of compromised immunological response usually associated with HIV on taeniosis, CC or NCC was diluted. An additional HIV+/HAART– group would have provided more detailed information on a possible influence of HAART (or the initiation of it) on the prevalence of T/CC/NCC and its clinical characteristics. However, recruitment of a sufficient number of HIV+ people that do not receive HAART was not possible within the study period due to national ethical reasons. So far, severe progressive and fatal NCC cases in HIV+ individuals were mostly described in patients who were not receiving HAART [7, 62, 63]. However, a recent cross-sectional study of a group of 601 HIV+ individuals conducted in Mozambique could not detect a difference in the presence of CC-Ab between HIV+ patients who were taking HAART and those who were not, nor was there a correlation between HAART duration and CC-Ab titers [31].

## Conclusions

In conclusion, we did not detect any difference in prevalence and manifestation of taeniosis, CC and NCC between matched HIV+ and HIV– individuals in a TSOL endemic area of northern Tanzania. However, the herein presented data on TSOL/HIV co-infection obtained in a comparative study design are the first of its kind. This study clearly points out that further large-scale studies are urgently required to re-examine TSOL infection in HIV+ individuals and in patients who are and are not on HAART, as well as NCC progression in HIV+ individuals.

## Additional files

Additional file 1: Multilingual abstracts in the six official working languages of the United Nations. (PDF 837 kb)
Additional file 2: Table S1. Clinical data, data on hygiene and eating habits, medication, family history and diagnostic data of TSOL+ individuals (*n* = 10) of the initially recruited study population. (DOC 74 kb)

## Abbreviations

Ab: Antibody
Ag: Antigen
AIDS: Acquired immunodeficiency syndrome
CC: Cysticercosis
CD4^+^: CD4^+^ T-lymphocytes
CDC: Centers for Disease Control and Prevention
CI: Confidence interval
CNS: Central nerval system
CT: Computed tomography
CTC: HIV care and treatment centre
CWGESA: Cysticercosis Working Group in Eastern and Southern Africa
ELISA: Enzyme linked immunosorbent assay
HAART: Highly active antiretroviral therapy
HIV: Human immunodeficiency virus
HLH: Haydom Lutheran Hospital
IgG: Immunoglobulin G
LLGP-EITB: Lentin-lectin-glycoprotein-enzyme linked immunoelectrotransfer blot
mPPR: Matched prevalence proportion ratios
MRI: Magnetic resonance imaging
MUHAS: Muhimbili University of Health and Allied Sciences
NCC: Neurocysticercosis
NIMR: National Institute for Medical Research
P: Patient number
PNS: Peripheral nervous system
PWE: People with epilepsy
THMIS: Tanzania HIV/AIDS and Malaria Indicator Survey
TSOL: *Taenia solium* infection
TUM: Technical University Munich
WHO: World Health Organization

## References

[1] Roman G (2000). A proposal to declare neurocysticercosis an international reportable disease. Bull World Health Organ.

[2] Del Brutto OH. Neurocysticercosis: a review. Sci World J. 2012;2012:159821. 10.1100/2012/159821 PMC3261519 22312322

[3] Carabin H, Ndimubanzi PC, Budke CM, Nguyen H, Qian Y, Cowan LD (2011). Clinical manifestations associated with neurocysticercosis: a systematic review. PLoS Negl Trop Dis.

[4] Winkler AS (2012). Neurocysticercosis in sub-Saharan Africa: a review of prevalence, clinical characteristics, diagnosis, and management. Pathog Glob Health.

[5] Almeida SM (2011). Quality of life assessment in patients with neurocysticercosis. J Community Health.

[6] Carabin H, Krecek RC, Cowan LD, Michael L, Foyaca-Sibat H, Nash T (2006). Estimation of the cost of *Taenia solium* cysticercosis in Eastern Cape Province, South Africa. Trop Med Int Health.

[7] Praet N, Speybroeck N, Manzanedo R, Berkvens D, Nsame Nforninwe D, Zoli A (2009). The disease burden of *Taenia solium* cysticercosis in Cameroon. PLoS Negl Trop Dis.

[8] Ndimubanzi PC, Carabin H, Budke CM, Nguyen H, Qian YJ, Rainwater E (2010). A systematic review of the frequency of neurocysticercosis with a focus on people with epilepsy. PLoS Negl Trop Dis.

[9] Winkler AS, Blocher J, Auer H, Gotwald T, Matuja W, Schmutzhard E (2009). Epilepsy and neurocysticercosis in rural Tanzania – an imaging study. Epilepsia.

[10] Del Brutto OH (2005). Diagnostic criteria for neurocysticercosis, revisited. Pathog Glob Health.

[11] Mwape KE, Blocher J, Wiefek J, Schmidt K, Dorny P, Praet N (2015). Prevalence of neurocysticercosis in people with epilepsy in the Eastern province of Zambia. PLoS Negl Trop Dis.

[12] World Health Organization: Winkler AS and Richter H (2015) Landscape analysis: management of neurocysticercosis with an emphasis on low- and middle-income countries. WHO/HTM/NTD/NZD/2015.05. Available: http://www.who.int/taeniasis/resources/who_htm_ntd_nzd_2015.05/en/. Accessed 23 Nov 2016.

[13] Gilman RH, Del Brutto OH, García HH, Martínez M (2000). Prevalence of taeniosis among patients with neurocysticercosis is related to severity of infection. Neurology.

[14] Thomas LF (2013). Epidemiology of *Taenia solium* cysticercosis in western Kenya.

[15] Mwape KE, Phiri IK, Praet N, Muma JB, Zulu G, Van den Bossche P (2012). *Taenia solium* infections in a rural area of Eastern Zambia-a community based study. PLoS Negl Trop Dis.

[16] Mwape KE, Phiri IK, Praet N, Speybroeck N, Muma JB, Dorny P (2013). The incidence of human cysticercosis in a rural community of Eastern Zambia. PLoS Negl Trop Dis.

[17] Mwanjali G, Kihamia C, Kakoko DVC, Lekule F, Ngowi H, Johansen MV (2012). Prevalence and risk factors associated with human *Taenia solium* infections in Mbozi district, Mbeya region, Tanzania. PLoS Negl Trop Dis.

[18] Braae UC, Magnussen P, Ndawi B, Harrison W, Lekule F, Johansen MV (2015). Effect of repeated mass drug administration with praziquantel and track and treat of taeniosis cases on the prevalence of taeniosis in *Taenia solium* endemic rural communities of Tanzania. Acta Trop.

[19] Phiri IK, Ngowi H, Afonso S, Matenga E, Boa M, Mukaratirwa S (2003). The emergence of *Taenia solium* cysticercosis in Eastern and Southern Africa as a serious agricultural problem and public health risk. Acta Trop.

[20] World Health Organization, Regional office for Africa. Overview: HIV/AIDS. 2015. Available: http://www.afro.who.int/en/hiv/overview.html. Accessed 23 Nov 2016.

[21] Murray CJ, Ortblad KF, Guinovart C, Lim SS, Wolock TM, Roberts DA (2014). Global, regional, and national incidence and mortality for HIV, tuberculosis, and malaria during 1990–2013: a systematic analysis for the global burden of disease study 2013. Lancet.

[22] Simon GG (2015). Impacts of neglected tropical disease on incidence and progression of HIV/AIDS, tuberculosis, and malaria: scientific links. Int J Infect Dis.

[23] Idemyor V (2007). Human immune deficiency virus (HIV) and malaria interaction in sub-Saharan Africa: The collision of two titans. HIV Clin Trials.

[24] Fincham JE, Markus MB, Adams VJ (2003). Could control of soil-transmitted helminthic infection influence the HIV/AIDS pandemic. Acta Trop.

[25] Kojic EM, White AC (2003). A positive enzyme-linked immunoelectrotransfer blot assay result for a patient without evidence of cysticercosis. Clin Infect Dis.

[26] Brown M, Mwa PA, Kaleebu P, Elliott AM (2006). Helminths and HIV infection: epidemiological observations on immunological hypotheses. Parasite Immunol.

[27] Delobel P, Signate A, El Guedj M, Couppie P, Gueye M, Smadja D (2004). Unusual form of neurocysticercosis in patients with HIV infection. Eur J Neurol.

[28] Prasad S, MacGregor RR, Tebas P, Rodriguez LB, Bustos JA, White AC (2006). Management of potential neurocysticercosis in patient with HIV infection. Clin Infect Dis.

[29] Foyaca-Sibat H, Ibanez-Valdes L (2003). Intraventricular neurocysticercosis in HIV positive patients. Internet J Neurol.

[30] Foyaca-Sibat H, Ibanez-Valdes L (2003). Neurocysticercosis in HIV-positive patients. Internet J Inf Dis.

[31] Noormahomed EV, Nhacupe N, Mascaró-Lazcano C, Mauaie MN, Buene T, Funzamo CA (2014). A cross-sectional serological study of cysticercosis, schistosomiasis, toxocariasis and echinococcosis in HIV-1 infected people in Beira, Mozambique. PLoS Negl Trop Dis.

[32] Serpa JA, Moran A, Goodman JC, Giordano TP, White AC (2007). Neurocysticercosis in the HIV Era: A case report and review of literature. Am J Trop Med Hyg.

[33] Jessurun J, Barrón-Rodríguez LP, Fernández-Tinoco G, Hernández-Avila M (1992). The prevalence of invasive amebiasis is not increased in patients with AIDS. AIDS.

[34] Shelburne SA, Montes M, Hamill RJ (2006). Immune reconstitution inflammatory syndrome: more answers, more questions. J Antimicrob Chemother.

[35] Haydom Lutheran Hospital. Statistics 2012. 2014. http://www.haydom.com/?page_id=1389. Accessed 23 Nov 2016.

[36] The United Republic of Tanzania, Ministry of Agriculture, Food Security and Cooperatives. National sample census of agriculture 2007/2008 Volume Vu: Regional report Manyara Region. 2012. Available: http://www.nbs.go.tz. Accessed 23 Nov 2016.

[37] The United Republic of Tanzania, Ministry of Agriculture, Food Security and Cooperatives. National sample census of agriculture 2007/2008 Volume Vm: Regional report Singida Region. 2012. Available: http://www.nbs.go.tz. Accessed 23 Nov 2016.

[38] The United Republic of Tanzania, National Bureau of Statistics (NBS) and Office of the Chief Government Statistician (OCGS-Zanzibar). HIV/AIDS and malaria indicator survey 2011–2012 (THMIS). 2013. Available: http://www.nbs.go.tz. Accessed 23 Nov 2016.

[39] Yahya-Malima KI, Matee MI, Evjen-Olsen B, Fylkesnes K (2007). High potential of escalating HIV transmission in a low prevalence setting in rural Tanzania. BMC Public Health.

[40] World Health Organization (WHO). WHO case definitions of HIV for surveillance and revised clinical staging and immunological classification of HIV-related disease in adults and children. Geneva: WHO; 2006. ISBN: 9789241595629.

[41] Tsang VC, Brand JA, Boyer AE (1989). An enzyme-linked immunoelectrotransfer blot assay and glycoprotein antigens for diagnosing human cysticercosis (*Taenia solium*). J Infect Dis.

[42] Wilson M, Bryan RT, Fried JA, Ware DA, Schantz PM (1991). Clinical evaluation of the cysticercosis enzyme-linked immunoelectrotransfer blot inpatients with neurocysticercosis. J Infect Dis.

[43] Noh J, Rodriguez S, Lee YM, Handali S, Gonzalez AE, Gilman RH (2014). Recombinant protein- and synthetic peptide-based immunoblot test for diagnosis of neurocysticercosis. J Clin Microbiol.

[44] Dorny P, Phiri IK, Vercruysse J, Gabriel S, Willingham AL, Brandt J (2004). A Bayesian approach for estimating values for prevalence and diagnostic test characteristics of porcine cysticercosis. Int J Parasitol.

[45] Wilkins PP, Allan JC, Verastegui M, Acosta M, Eason AG, Garcia HH (1999). Development of a serologic assay to detect *Taenia solium* taeniasis. Am J Trop Med Hyg.

[46] Levine MZ, Calderón JC, Wilkins PP, Lane WS, Asara JM, Hancock K (2007). Characterization, cloning, and expression of two diagnostic antigens for *Taenia solium* tapeworm infection. J Parasitol.

[47] Nash TE, Del Brutto OH, Butman JA, Corona T, Delgado-Escueta A, Duron RM (2004). Calcific neurocysticercosis and epileptogenesis. Neurology.

[48] CWGESA Action Plan. Available: http://ivs.ku.dk/english/research/about_parasitology_and_aquatic_diseases/parasitic_zoonoses/cwgesa. Accessed 23 Nov 2016.

[49] Guezala MC, Rodriguez S, Zamora H, Garcia HH, Gonzalez AE (2009). Development of a species-specific coproantigen ELISA for human *Taenia solium* taeniosis. Am J Trop Med Hyg.

[50] Kamuyu G, Bottomley C, Mageto J, Lowe B, Wilkins PP, Noh JC (2014). Exposure to multiple parasites is associated with the prevalence of active convulsive epilepsy in sub-Saharan Africa. PLoS Negl Trop Dis.

[51] Mwang’onde BJ, Nkwengulila G, Chacha M (2012). The serological survey for human cysticercosis prevalence in Mbulu district, Tanzania. AIDS.

[52] Ngowi HA, Kassuku AA, Carabin H, Mlangwa JE, Mlozi MR, Mbilinyi BP (2010). Spatial clustering of porcine cysticercosis in Mbulu district, northern Tanzania. PLoS Negl Trop Dis.

[53] Kanobana K, Praet N, Kabwe C, Dorny P, Lukanu P, Madina J (2011). High prevalence of *Taenia solium* cysticercosis in a village community of Bas-Congo, Democratic Republic of Congo. Int J Parasitol.

[54] Carabin H, Millogo A, Cissé A, Gabriël S, Sahlu I, Dorny P (2015). Prevalence of and factors associated with human cysticercosis in 60 villages in three provinces of Burkina Faso. PLoS Negl Trop Dis.

[55] Coral-Almeida M, Gabriël S, Abatih EN, Praet N, Benitez W, Dorny P (2015). *Taenia solium* human cysticercosis: a systematic review of sero-epidemiological data from endemic zones around the world. PLoS Negl Trop Dis.

[56] Parija SC, Gireesh AR (2009). A serological study of cysticercosis in patients with HIV. Rev Inst Med Trop Sao Paulo.

[57] Schantz PM, Sarti E, Plancarte A, Wilson M, Criales JL, Roberts J (1994). Community-based epidemiological investigations of cysticercosis due to *Taenia solium*: comparison of serological screening tests and clinical findings in two populations in Mexico. Clin Infect Dis.

[58] Walson JL, Herrin BR, John-Stewart G. Deworming helminth co-infected individuals for delaying HIV disease progression. Cochrane Database Syst Rev. 2009;(3):CD006419. 10.1002/14651858.CD006419.pub3 PMC2871762 19588389

[59] Gomez Morales MA, Atzori C, Ludovisi A, Rossi P, Scaglia M, Pozio E (1995). Opportunistic and non-opportunistic parasites in HIV-positive and negative patients with diarrhoea in Tanzania. Trop Med Parasitol.

[60] Lindo JF, Dubon JM, Ager AL, de Gourville EM, Solo-Gabriele H, Klaskala WI (1998). Intestinal parasitic infections in human immunodeficiency virus (HIV)-positive and HIV-negative individuals in San Pedro Sula, Honduras. Am J Trop Med Hyg.

[61] Mechain B, Garin YJF, Robert-Gangneux F, Dupouy-Camet J, Derouin F (2000). Lack of utility of specific immunoglobulin G antibody avidity for serodiagnosis of reactivated toxoplasmosis in immunocompromised patients. Clin Diagn Lab Immunol.

[62] Ramos JM, Masia M, Padilla S, Bernal E, Martin-Hidalgo A, Gutiérrez F (2007). Fatal infection due to larval cysts of cestodes (neurocysticercosis and hydatid disease) in human immunodeficiency virus (HIV) infected patients in Spain: report of two cases. Scand J Infect Dis.

[63] Wolday D, Mayaan S, Mariam ZG, Berhe N, Seboxa T, Britton S (2002). Treatment of intestinal worms is associated with decreased HIV plasma viral load. J Acquir Immune Defic Syndr.

