# Supplementary material for: Association between Taenia solium infection and HIV/AIDS in northern Tanzania: a matched cross sectional-study
Source: Infect Dis Poverty. 2016 Dec 1;5:111. doi: 10.1186/s40249-016-0209-7 (PMC5131417; doi:10.1186/s40249-016-0209-7)
Supplement: Additional file 1: — Multilingual abstracts in the six official working languages of the United Nations. (PDF 837 kb) [file 40249_2016_209_MOESM1_ESM.pdf]

Translation of the abstract into the five official working languages of the United Nations

الترابط بين عدوى *الشريطية الوحيدة* وفيروس العوز المناعي البشري/نقص المناعة المكتسب في شمالي تنزانيا: دراسة مقطعية مقارنة

فيرونيكا شميت، كريستيان كوزيتس، كارل-هاينز هربنجر، إيلين كارابان، برنار نگاوي، إزرا نامان، باتريشا بي ويلكنز، جون نوه، ويليام ماتوجا، آندري سلفيا ونكلر

خلاصة

الخلفية: تواتر *الشريطية الوحيدة*، وهي دودة طفيلية حيوانية المصدر، متصاعد في عدد من بلدان أفريقية جنوب الصحراء التي ينتشر فيها أيضًا فيروس العوز المناعي البشري (HIV). لكن المعروف عن تفاعل هاتين العدويَين قليل. كان الهدف من هذه الدراسة مقارنة نسبة المصابين بفيروس العوز المناعي البشري (+) وغير المصابين به (-) المصابين *بالشريطية الوحيدة* (TSOL) والذين تتجلى فيهم مظاهر سريرية وعصبية لداء الكيسات المذنَّبة (CC).

المناهج: في شماليّ تنزانيا، تم ترغيب 170 شخصًا مصابًا بفيروس العوز المناعي البشري و170 مرجعًا غير مصابين به متطابقين في النوع والعمر والقرية الأصل. تم إجراء اختبارات لتصنيف مراحل فيروس العوز المناعي البشري واختبارات سيرولوجية للفيروس بحثًا عن الأجسام المضادّة (Ab) والمستضدّات (Ag). تم تحديد داء الكيسات المذنَّبة العصبي (NCC) بالتصوير المقطعي المُحوسَب (CT) باستخدام المعايير التشخيصية القياسية. وتم تأكيد المظاهر العصبية بفحص عصبي قياسي. كما تم جمع البيانات الديموغرافية والسريرية وبيانات التصوير العصبي. تم أيضًا تسجيل أعداد خلايا كتلة التمايز 4 ($CD4^+$) بالإضافة إلى معلومات العلاج شديد الفاعلية للفيروسات الارتدادية (HAART).

النتائج: لم يتم الكشف عن أية اختلافات هامة بين الأفراد المصابين بفيروس العوز المناعي البشري والأفراد غير المصابين به من ناحية انتشار الأجسام المضادّة لداء الشريطيات (0.6% مقابل 1.2%) والأجسام المضادّة لداء الكيسات المذنَّبة (2.4% مقابل 2.4%) ومستضدّات داء الكيسات المذنَّبة (0.6% مقابل 0.0%) في الأمصال. وتم الكشف عن ست حالات مصابة بداء الكيسات المذنَّبة العصبي (3 منها مصابة بفيروس العوز المناعي البشري و3 منها بريئة منه) في مجموعة المشاركين المطابقين. ظهر على فردين (واحد مصاب بفيروس العوز المناعي البشري وواحد بريء منه) نوبات صداع كعرض أساسي لداء الكيسات المذنَّبة العصبي بينما كان أربعة مصابين بداء الكيسات المذنَّبة العصبي دون أعراض. وفي المجموعة المصابة بفيروس العوز المناعي البشري، لم تكن الشريطية الوحيدة مرتبطة بأعداد خلايا كتلة التمايز 4^+ أو مدة العلاج شديد الفاعلية للفيروسات الارتدادية أو مرحلة فيروس العوز المناعي البشري.

الخاتمة: كشفت هذه الدراسة عن معدل لانتشار داء الشريطيات وداء الكيسات المذنَّبة وداء الكيسات المذنَّبة العصبي أقل من المسجّل في المنطقة حتى تاريخه. قد يكون انخفاض مستوى العدوى هذا ناتجًا عن انعدام للقدرة على العثور على ترابطات مقطعية بين حالة فيروس العوز المناعي البشري وعدوى الشريطية الوحيدة أو داء الكيسات المذنَّبة العصبي. وستتطلب الدراسات المستقبلية التي يتم إجراؤها في هذه المنطقة عينات أكبر لاستنتاج ما إذا كان فيروس العوز المناعي البشري يؤثر على طريقة ظهور داء الكيسات المذنَّبة العصبي.

Translated from English version into Arabic by arwam, through

TRANSLATORS
WITHOUT BORDERS

坦桑尼亚北部地区猪带绦虫感染和 HIV/AIDS 相关性研究：一项配对横断面调查

Veronika Schmidt, Christian Kositz, Karl-Heinz Herbinger, Hélène Carabin, Bernard Ngowi, Ezra Naman, Patricia P. Wilkins, John Noh, William Matuja, Andrea Sylvia Winkler

摘要

引言：撒哈拉以南的一些非洲国家猪带绦虫感染逐渐增加，而且这些国家的 HIV/AIDS 感染率也很高。但是两者间的相互关系知之甚少。本研究旨在通过比较感染猪带绦虫人群中的 HIV 阳性和阴性个体，描述囊尾蚴病神经系统表现和临床特征。

方法：在坦桑尼亚北部地区，招募在年龄、性别和籍贯（村）相匹配的 170 例 HIV 阳性者和 170 例 HIV 阴性者。对所有人员进行艾滋病分期和针对 TSOL 抗体(Ab)和抗原(Ag)的血清学检测。应用 CT 根据标准诊断标准对神经囊尾蚴病进行诊断，采用标准神经系统检查神经系统表现。此外，同时收集人口统计学、临床和神经影像数据。并进一步记录 $CD4^+$细胞计数以及高效抗逆转录病毒治疗的信息。

结果：HIV 阳性和阴性者带绦虫抗体(0.6 % vs 1.2 %)，CC-Ab (2.4 % vs 2.4 %)和 CC-Ag (0.6 % vs 0.0 %)血清阳性率间的差异无统计学意义。在配对的招募人员中，发现 6 例神经囊尾蚴病（3 例 HIV 阳性和 3 例 HIV 阴性），其中 2 例（1 例 HIV 阳性和 1 例 HIV 阴性）出现以头痛为主的神经囊尾蚴病症状，另外 4 例为无症状患者。HIV 阳性组中，TSOL 与 $CD4^+$细胞计数、高效抗逆转录病毒治疗时间和 HIV 分期无相关性。

结论：本研究发现猪带绦虫病、神经囊尾蚴病和囊尾蚴病的患病率低于目前该地区报道的情况。低水平感染也许是导致 HIV 状态与猪带绦虫感染和神经囊尾蚴病横断面研究无相关性的原因。在将来的研究中需要进一步扩大样本来确定 HIV 能够影响脑囊虫病的特征。

Translated from English version into Chinese by Xin-Yu Feng, edited by Pin Yang

CDC CHINESE CENTER FOR DISEASE CONTROL AND PREVENTION
NATIONAL INSTITUTE OF PARASITIC DISEASES

Association entre les infestations par *Taenia solium* et le VIH/SIDA dans le nord de la Tanzanie : une étude transversale appariée

Veronika Schmidt, Christian Kositz, Karl-Heinz Herbinger, Hélène Carabin, Bernard Ngowi, Ezra Naman, Patricia P. Wilkins, John Noh, William Matuja, Andrea Sylvia Winkler

Résumé

Contexte : La fréquence de *Taenia solium*, un helminthe zoonotique, est en augmentation dans de nombreux pays d'Afrique subsaharienne, où la prévalence du virus de l'immunodéficience humaine (VIH) est également élevée. Cependant, les interactions entre ces deux infections sont très mal connues. Le but de cette étude est de comparer les proportions d'individus séropositifs (+) et séronégatifs (-) pour le VIH infestés par *Taenia solium* et qui présentent des manifestations cliniques et neurologiques de cysticercose (CC).

Méthodes : Nous avons recruté 170 individus VIH+ et 170 témoins VIH- appariés par sexe, âge et village d'origine dans le nord de la Tanzanie. Le stade d'infection par le VIH a été déterminé et des tests sérologiques de recherche des anticorps (Ac) et de l'antigène (Ag) de *T. solium* ont été effectués. La neurocysticercose (NCC) a été mise en évidence par tomodensitométrie (TDM) en utilisant des critères de diagnostic standard. Les manifestations neurologiques ont été confirmées par un examen neurologique standard. Des données démographiques, cliniques et de neuroimagerie

ont également été collectées. La numération des CD4^{+} ainsi que des informations sur les traitements antirétroviraux à haute activité (HAART) ont également été relevées.

Résultats : Aucune différence significative n’a été relevée entre les individus VIH+ et VIH- concernant la séroprévalence des anticorps anti-ténia (0,6 % contre 1,2 %) et anti-CC (2,4 % contre 2,4 %) ainsi que de l’antigène de cysticerque (0,6 % contre 0,0 %). Six cas de NCC (3 VIH+ et 3 VIH–) ont été détectés dans le groupe de participants appariés. Elle se manifestait principalement par des maux de tête chez deux sujets (1 VIH+ et 1 VIH–) et était asymptomatique chez les quatre autres. Parmi le groupe VIH+, la présence de *T. solium* n'a pas été associée à la numération de CD4^{+}, à la durée du HAART ni au stade d’infection par le VIH.

Conclusions : Notre étude a trouvé une prévalence de la taeniose, de la cysticercose et de la neurocysticercose inférieure à celle reportée dans la région à ce jour. Ce faible niveau d'infestation peut nous avoir empêchés de trouver des associations transversales entre le statut VIH et l’infestation par *T. solium* ou la neurocysticercose. Les études futures menées dans cette région devront utiliser des échantillons de plus grande taille pour déterminer si le VIH influence la façon dont la NCC se manifeste.

Translated from English version into French by Suzanne Assenat, through

TRANSLATORS WITHOUT BORDERS

Взаимосвязь между *свиным цепнем* и ВИЧ/СПИД в северной Танзании: попарное кросс-секционное исследование

Вероника Шмидт, Кристиан Коситз, Карл-Хайнц Хёрбингер, Элен Карабин, Бернар Нгови, Эзра Наман, Патисия П. Уилкинс, Джон Нох, Уильям Матуха, Андреа Сильвия Винклер

Краткое описание

Предпосылки и цель исследования: Частота случаев заболеваний ***свиным цепнем***, зоонозным гельминтозом увеличивается во многих африканских странах, расположенных к югу от Сахары, где распространенность вируса иммунодефицита человека (ВИЧ) также высока. Тем не менее, мало известно о том, как эти две инфекции взаимодействуют между собой. Целью данного исследования было сравнение доли ВИЧ-инфицированных положительных (+) и отрицательных (-), инфицированных свиным цепнем (TSOL), и у которых наблюдаются клинические и неврологические проявлениями цистицеркоза (CC).

Методы исследования: В северной части Танзании в исследовании приняли участие 170 ВИЧ-инфицированных лиц, а также парные 170 ВИЧ-инфицированные для сравнения по полу, возрасту, месту проживания и происхождению. Были проведены стадирование ВИЧ заболевания, а также серологические анализы на свиной цепень (TSOL), антитела (Ab) и антигены (Ag). Нейроцистицеркоз (NCC) определяли с помощью компьютерной томографии (КТ) с использованием стандартных диагностических критериев. Неврологические проявления были подтверждены с помощью стандартного неврологического обследования.

Кроме того, были собраны демографические, клинические, а также данные нейровизуализационного обследования. Кроме того, был проведен подсчет клеток CD4 +, а также высокоактивной антиретровирусной терапия (ВААРВТ).
Результаты: Никаких существенных различий между ВИЧ + и ВИЧ-лиц, имеющих серопревалентность тениоза-Ab (0,6% против 1,2%), не было обнаружено: CC-Ab (2,4% против 2,4%) и CC-Ag (0,6% против 0,0%). В общей сложности в группе парных участников были обнаружены шесть случаев нейроцистицеркоза (NCC) (3 ВИЧ + и 3 ВИЧ-). Два человека (1 с ВИЧ + 1 с ВИЧ-) жаловались на головные боли, как на главный симптом нейроцистицеркоза (NCC), а четыре имели бессимптомное заболевание. Среди группы больных в ВИЧ + свиной цепень (TSOL) не имел связи с количеством клеток $CD4^+$ продолжительности высокоактивной антиретровирусной терапии (ВААРВТ), или стадии ВИЧ-инфекции.
Вывод: Результаты данного исследования показали более низкую распространенность инфекции тениоза, цистицеркоза (CC) и нейроцистицеркоза (NCC), чем сообщалось в регионе на сегодняшний день. Такой низкий уровень инфекции может сделать невозможным обнаружить взаимосвязь между ВИЧ-статусом, свиным цепнем (TSOL) или нейроцистицеркозом (NCC). В будущем для исследования потребуется большее количество образцов, взятых в этом регионе, чтобы определить: влияет ли ВИЧ на способ проявления нейроцистицеркоза (NCC).

Translated from English version into Russian by Gulnara Krokhaleva, through

TRANSLATORS
WITHOUT BORDERS

Relación entre la infección por *Taenia solium* y el VIH/SIDA en el norte de Tanzania: estudio transversal combinado

Veronika Schmidt, Christian Kositz, Karl-Heinz Herbinger, Hélène Carabin, Bernard Ngowi, Ezra Naman, Patricia P. Wilkins, John Noh, William Matuja, Andrea Sylvia Winkler

Resumen

Contextualización: la recurrencia de la *Taenia solium*, un parásito zoonótico, está aumentando en muchos países del África subsahariana, donde la prevalencia del virus de inmunodeficiencia humana (VIH) también es alta. Sin embargo, se sabe poco sobre cómo interactúan estas dos infecciones. El objetivo de este estudio era comparar la proporción de individuos con VIH positivo (+) y negativo (-) infectados con *Taenia solium* (T. solium) y que presentan manifestaciones clínicas y neurológicas de cisticercosis (CC).
Método: en el norte de Tanzania, se reclutaron 170 individuos VIH+ y 170 individuos VIH- de control emparejados por sexo, edad y pueblo de origen. Se realizaron exámenes clínicos de VIH y pruebas serológicas para la detección de anticuerpos (Ac) y antígenos (Ag). La neurocisticercosis (NCC) se determinó mediante tomografía computarizada (TC) utilizando criterios diagnósticos

estándar. Se confirmaron las manifestaciones neurológicas mediante un examen neurológico estándar. Además, se recogieron datos demográficos, clínicos y de neuroimagen. Asimismo, se tomó nota de los recuentos de células $CD4^+$ y de información sobre terapia antirretroviral de gran actividad (HAART, por sus siglas en inglés).
Resultados: no se observaron diferencias significativas entre los individuos VIH+ y VIH- en relación con la seroprevalencia de anticuerpos de teniasis (0.6% vs. 1,2%), de anticuerpos de CC (2,4% vs. 2,4%) y de antígenos de CC (0,6 % vs 0,0 %). Se detectaron un total de seis casos de NCC (3 VIH+ y 3 VIH-) en el grupo de participantes emparejados. Dos individuos (1 VIH+ y 1 VIH-) presentaron cefaleas como síntoma principal de la NCC y cuatro presentaron NCC asintomática. En el grupo VIH+, la T. solium no estaba relacionada con el recuento de células $CD4^+$, la duración de la terapia HAART o el estadio de la infección por VIH.
Conclusiones: este estudio encontró una prevalencia de la teniasis, la CC y la NCC menor a la que se había comunicado que existía en la región hasta la fecha. Este bajo nivel de infección puede haber sido la causa de la imposibilidad de encontrar relaciones transversales entre la seropositividad y la infección por T. solium o NCC. Es necesario contar con un tamaño de muestra mayor en los futuros estudios que se realicen la zona para concluir si el VIH influye en la manera en que se manifiesta la NCC.

Translated from English version into Spanish by Maria Alejandra Aguada, through

TRANSLATORS
WITHOUT BORDERS
