# Supplementary material for: Association between Taenia solium infection and HIV/AIDS in northern Tanzania: a matched cross sectional-study
Source: Infect Dis Poverty. 2016 Dec 1;5:111. doi: 10.1186/s40249-016-0209-7 (PMC5131417; doi:10.1186/s40249-016-0209-7)
Supplement: Additional file 2: Table S1. — Clinical data, data on hygiene and eating habits, medication, family history and diagnostic data of TSOL+ individuals (n = 10) of the initially recruited study population. (DOC 74 kb) [file 40249_2016_209_MOESM2_ESM.doc]

**Supporting table 1.** Clinical characteristics, data on hygiene and eating habits, medication, family history and diagnostic data of TSOL individuals (*n* = 10) of the initially recruited population.

| Variables | TSOL+/HIV+ (4) | | TSOL+/HIV– (6) | | TSOL+ Total (10) | |
| --- | --- | --- | --- | --- | --- | --- |
|  | *n* | % | *n* | % | *n* | % |
| History of acute headaches |  |  |  |  |  |  |
| Yes | 1 | 25.0 | 0 | 0.0 | 1 | 10.0 |
| History of chronic headaches |  |  |  |  |  |  |
| Yes | 0 | 0.0 | 2 | 34.0 | 2 | 20.0 |
| Past or present epileptic seizures |  |  |  |  |  |  |
| Yes | 0 | 0.0 | 2 | 34.0 | 2 | 20.0 |
| Past psychiatric disorders |  |  |  |  |  |  |
| Yes | 0 | 0.0 | 2 | 34.0 | 2 | 20.0 |
| Current CNS symptoms |  |  |  |  |  |  |
| Yes | 0 | 0.0 | 1 | 17.0 | 1 | 10.0 |
| Current PNS symptoms |  |  |  |  |  |  |
| Yes | 0 | 0.0 | 0 | 0.0 | 0 | 0.0 |
| HAART duration |  | | | | | |
| Up to 2 years | 1 | 15.0 | n.a. | n.a. | 1 | 15.0 |
| 3 - 4 years | 3 | 75.0 | n.a. | n.a. | 3 | 75.0 |
| 5 - 6 years | 0 | 0.0 | n.a. | n.a. | 0 | 0.0 |
| Up to 9 years | 0 | 0.0 | n.a. | n.a. | 0 | 0.0 |
| Not known | 0 | 0.0 | n.a. | n.a. | 0 | 0.0 |
| CD4^+^ counts |  |  |  |  |  |  |
| <200 | 1 | 25.0 | n.a. | n.a. | 1 | 25.0 |
| 201 - 350 | 1 | 25.0 | n.a. | n.a. | 1 | 25.0 |
| 351 - 500 | 1 | 25.0 | n.a. | n.a. | 1 | 25.0 |
| >500 | 1 | 25.0 | n.a. | n.a. | 1 | 25.0 |
| Opportunistic infections |  |  |  |  |  |  |
| Yes | 1 | 250 | n.a. | n.a. | 1 | 25.0 |
| CC-Ag |  |  |  |  |  |  |
| Positive | 1 | 25.0 | 1 | 17.0 | 2 | 20.0 |
| CC-Ab tested by LLGP-EITB |  |  |  |  |  |  |
| Positive | 3 | 75.0 | 6 | 100.0 | 9 | 90.0 |
| CC-Ab tested by rT24H-blot |  |  |  |  |  |  |
| Positive | 3 | 75.0 | 6 | 100.0 | 9 | 90.0 |
| T-Ab |  |  |  |  |  |  |
| Positive | 1 | 25.0 | 4 | 66.0 | 5 | 50.0 |
| NCC* |  |  |  |  |  |  |
| Positive | 4 | 100.0 | 5 | 83.0 | 9 | 90.0 |
| n.k. | 0 | 0.0 | 1 | 17.0 | 1 | 10.0 |
| Consumes pork |  |  |  |  |  |  |
| Yes | 4 | 100.0 | 6 | 100.0 | 10 | 100.0 |
| History of tapeworm carrier in family | | |  |  |  |  |
| Yes | 0 | 0.0 | 0 | 0.0 | 0 | 0.0 |
| Handwashing before eating |  |  |  |  |  |  |
| Yes | 0 | 0.0 | 0 | 0.0 | 0 | 0.0 |
| Anthelmintic treatment in the past year | | |  |  |  |  |
| Yes | 1 | 25.0 | 1 | 17.0 | 2 | 20.0 |

CNS: central nervous system, PNS: peripheral nervous system, HAART: highly active antiretroviral therapy, n.a.: not applicable, because in the group of HIV– these parameters were not tested and no HAART was taken, CD4+: CD4+ T-lymphocyte cell counts, CC: cysticercosis, Ag: antigen, Ab: antibody, LLGP-EITB: lentin-lectin glycoprotein electroimmunosorbent blot, n.k.: not known, because CT scan was not take due to refusal of patient.

* According to the revised diagnostic criteria proposed by Del Brutto [10].
